# Psychological empowerment and job satisfaction in nurses: A systematic review and meta-analysis

**DOI:** 10.3389/fpubh.2022.1022823

**Published:** 2022-11-11

**Authors:** Lihua Gu, Liping Wang, Biwei Pan

**Affiliations:** ^1^School of Nursing and Health, Shanghai Zhongqiao Vocational and Technical University, Shanghai, China; ^2^Department of Nursing and Home Economics, Shanghai Open University Yangpu Branch, Shanghai, China; ^3^Department of Interventional Oncology, Renji Hospital, Shanghai Jiao Tong University School of Medicine, Shanghai, China

**Keywords:** job satisfaction, meta-analysis, nurses, psychological empowerment, psychological burden

## Abstract

**Background:**

Psychological empowerment is generally understood to be associated with job satisfaction among nurses. However, recently published literature has questioned this association.

**Objective:**

We aimed to systematically investigate through a meta-analysis the association of psychological empowerment with job satisfaction among nurses.

**Methods:**

PubMed, Medline, Cochrane Library, PsycInfo, and Embase were used to search targeted studies from conception to 20 January 2022. The correlation coefficients of each study were extracted and converted into Fisher's Z. Finally, pooled *r* was calculated by Fisher's Z and standard error (SE).

**Results:**

A total of 28 studies encompassing 27 articles with 7,664 registered nurses were included. The pooled correlation coefficient between psychological empowerment and job satisfaction was 0.55. Subgroup analyses were conducted according to ethnicity, and the correlation in the Asian participants (*P* < 0.01) was significantly stronger (*P* < 0.05) than that of the Caucasian nurses (*P* < 0.01).

**Conclusions:**

Psychological empowerment is strongly correlated to job satisfaction for registered nurses, especially among those from Asian countries. Interventions designed for psychological empowerment could be one of the strategies to promote the retention of nurses. Nonetheless, additional future studies are essential for more investigations.

## Introduction

Several factors, such as complications in nursing practice, workload, and workplace conditions, might individually or in combination lead to internal frustration or emotionally exhaust the nurses (1), and lead to a nursing shortage (1). The lack of an adequate nursing population aggravates psychological and physical health problems, which ultimately results in emotional exhaustion among nurses. Job satisfaction for nurses could be influenced by their working environment, personal characteristics, and organizational factors (1).

Despite the shortage of human and financial resources, the management of nurses must maintain high-quality standards regarding patient care and job satisfaction. Psychological empowerment mainly focuses on the nurses' workplace and is deemed as a strategy to prevent emotional exhaustion. However, whether psychological empowerment is an internal incentive factor and can influence nurse job satisfaction and outcomes is yet controversial (2–5).

In 2018, a meta-analysis (6) focused on this aspect and emphasized the impact of psychological empowerment on job satisfaction in nurses. However, in recent years, several studies (7–9) have provided new evidence (10), indicating that psychological empowerment might not be associated with job satisfaction among nurses. Therefore, conducting a new meta-analysis by pooling the newly published literature may systematically review current published evidence and provide convincing findings on this topic. In this study, we aimed to further investigate the association between psychological empowerment and job satisfaction among nurses.

## Methods

This current study was performed following the Preferred Reporting Items for Systematic Reviews and Meta-analysis guidelines (PRISMA) statement.

### Literature identification

PubMed, Medline, Cochrane Library, PsycInfo, and Embase databases were used to search for studies from their inception to 20 January 2022. The search terms included: “nurse”, “nurses”, “empower”, “job satisfaction”, “work satisfaction”, “psychological empowerment”, and “empowerment”. We used these search strings as broadly as possible. Google Scholar was used for studies citing relevant studies.

### Eligibility criteria

Articles that addressed the topic of the impact of nurses' psychological empowerment initiatives on their job satisfaction were included. Studies that met the following criteria were included: (1) studies included nurses as participants; (2) studies explored the association of psychological empowerment with job satisfaction; (3) necessary data could be obtained or calculated.

Studies reporting on other types of empowerment and including non-registered nurses, such as nursing students, nursing assistants, and allied healthcare professionals, were excluded. Qualitative studies, reviews, case reports, comments, conference abstracts, and studies that were not available were also excluded.

### Data extraction

All studies obtained from the above databases were screened and assessed independently by two authors. All needed information was extracted using a standardized form by the two authors independently, and a consensus was reached on all items in discussion with a third author. For each included study, the authors extracted the characteristics of the studies (author, publishing year, country, and sample size), participant information (mean age and the proportion of females), constructs measured (the scale used, number of items, means, and reliability), and the correlation coefficients.

### Quality scoring of studies

The two authors used the published quality assessment and validity tool for correlational studies. A consensus was reached on all inconsistent items through a discussion with another author. The tool assessed the quality of the included studies using 13 items to score the study design, sample size, measurements of result, and statistical analysis. The studies were awarded a maximum of one point for each of the 12 items (prospective studies, probable sampling, proper sample size, sample drawn from multiple sites, anonymity protected, response rate more than 60%, reliable measure of outcome, an efficacious measure of outcomes, empowerment internal consistency, theoretical framework used, correlation analysis for multiple effects, and management of outliers addressed), and a maximum of two points for one item (a valid measure of empowerment). The detailed items are presented in Supplementary Table 1. The score ranged from 0 to 14 points, where 0–4 points indicated poor quality, 5–9 points meant medium quality, and 10–14 points indicated high quality.

### Analysis

The correlation coefficient (*r*) for each study was extracted and transformed to Fisher's Z and standard error (SE), and the final effect size was calculated as the pooled *r* and 95% confidence interval (CI) based on the random-effects model. Due to different ethnicities and cultural backgrounds, subgroup analysis was done according to the source of the study population (Caucasian group *vs*. Asian group).

The standard heterogeneity test based on the *I*^2^ statistic was used to assess the consistency of the effect sizes. Heterogeneity was deemed to be significant if *I*^2^ ≥ 50%. For analysis with significant heterogeneity, we examined the robustness of the pooled result by using the leave-one-out method. In addition, studies with low or medium-quality scores were separated from those with high quality for the sensitivity analysis. We assessed the publication bias using Begg's and Egger's weighted regression methods. The publication bias was performed by STATA 15.0 (Stata Corporation, College Station, TX, USA). A *P*-value of <0.05 was considered statistically significant.

### Ethics approval

No ethical approval and informed consent were required because the present study was a network meta-analysis of published studies.

## Results

### Study selection

**Figure 1:**
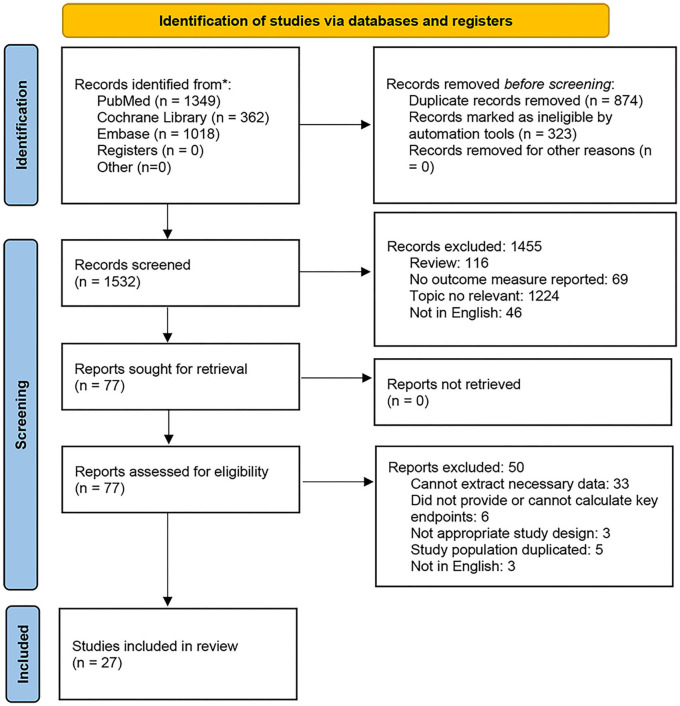
Flowchart of the study selection.

The flowchart of study retrieval and selection is displayed in Figure 1. In total, 2,729 studies were retrieved and 874 were excluded due to duplication among various datasets. Following the above-mentioned inclusion and exclusion criteria, 1,455 abstracts or titles were assessed initially. After reading 77 full-length manuscripts, ultimately, 27 articles were included. One of the included studies (11) was a comparative analysis of nurses in Malaysia and England and was classified by ethnicity.

### Study characteristics

A total of 28 studies were included in the present study and these were published between 1997 and 2019. The sample size of nurses included in these studies ranged from 56 to 1007, with a total of 7664 nurses. Most of the studies included clinically registered nurses and two nurse managers (3, 12), one school health nurse (13), nurses, midwives (4), and female caregivers (5). Most of the studies (85%) were cross-sectional. Ten of the included studies were conducted in Canada (3, 12, 14–21), eight in USA (2, 9, 19, 22–26), two each in China (13, 27) and Spain (8, 10), and one each in England (11), Malaysia (11), Ireland (4), Denmark (7), Sweden (5), and South Korea (28). The measures for job satisfaction were the “job satisfaction scale” or “general job satisfaction scale.” About 93% of the researchers assessed psychological empowerment using the “psychological empowerment scale” or “conditions for work effectiveness questionnaire.” Most of the participants were female, and the mean age of the participants was 28–50 years. The mean years of their nursing experience ranged from 1.17 to 29.22 years. Of the 28 studies, 14 of them reported a response rate of> 50%. The characteristics of the included studies and participants are summarized in Tables 1, 2.

**Table 1:** Characteristics of the included studies.

**Study included**	**Country**	**Study design**	**Subjects**	**Measures**	**Reliability Cronbach (α)**	**Analysis methods**
Morrison et al. (22)	USA	Cross-sectional	Registered nurses	Job satisfaction scale	0.72	Hierarchical regression analysis, correlation analysis
				PES	0.78	
Fuller et al. (23)	USA	Cross-sectional	Registered nurses	PES	0.70	Moderated multiple regression analysis
				Job satisfaction scale	0.89	
Laschinge et al. (14)	Canada	Cross-sectional	Registered nurses	PES	0.89	Path analysis, structural equation modeling analyses
				Job satisfaction scale	0.82	
				CWEQ-II	0.82	
Manojlovich et al. (15)	Canada	Cross-sectional	Registered nurses	CWEQ	0.95	Hierarchical regression correlation analyses
				PES	0.88	
				Job satisfaction scale	0.81	
Larrabee et al. (2)	USA	Cross-sectional	Registered nurses	WQI	0.95	Correlation analyses, multivariate regression analyses
				PES	0.91	
Laschinger et al. (16)	Canada	Cross-sectional	Registered nurses	Job satisfaction scale	0.78/0.84 ^a^	Structural equation modeling
				PES	0.87/0.89 ^a^	
				CWEQ-II	0.77/0.82 ^a^	
Laschinger et al. (16)	Canada	Cross-sectional	Nurse managers	Job satisfaction scale	0.84–0.88	Hierarchical multiple regression and correlation analyses
				PES	0.87–0.92	
				CWEQ-II	0.79–0.82	
Laschinger et al. (12)	Canada	Cross-sectional	Nurse managers	Job satisfaction scale	0.88	Structural equation modeling analyses
				PES	0.87–0.92	
				CWEQ-II	0.79–0.82	
Kostiwa et al. (24)	USA	Cross-sectional	Registered nurses	PES	0.83–0.87	Multiple linear regression analysis, correlation analysis
				Overall scale	0.93–0.94	
Tourangeau et al. (31)	Canada	Cross-sectional	Registered nurses	General job satisfaction scale	0.78	Bivariate regression analyses, stepwise regression modeling
				PES	0.86	
Ahmad et al. (11)	England and Malaysia	Cross-sectional	Registered nurses	General job satisfaction scale	0.78–0.90	Spearman's rank correlation analysis, multiple regression analysis
				PES	NA	
Chang et al. (13)	China	Cross-sectional	School health nurses	Job satisfaction scale	0.77	Linear regression, Path analysis, structural equation modeling
				PES	0.83	
				CWEQ-II	0.89	
Casey et al. (4)	Ireland	Cross-sectional	Nurses and midwives	Job Satisfaction Scale	0.79	Regression analysis and Spearman's rank correlation analysis
				PES	0.62–0.72	
				CWEQ-II	0.68–0.88	
Engstrom et al. (29)	Sweden	Cross-sectional	Female caregivers	PES	NA	Spearman's rank-order correlation analyses
				Job satisfaction scale	NA	
Sparks et al. (17)	USA	Cross-sectional	Registered nurses	Job satisfaction scale	NA	Correlations, chi-square test, general linear modeling procedures
				PES	0.79–0.85	
Wagner et al. (18)	Canada	Cross-sectional	Registered nurses	Job satisfaction scale	0.72	Pearson's chi-square analysis, structural equation model
				PES	0.62–0.72	
				CWEQ-II	0.78–0.81	
Cramer et al. (30)	USA	Mixed method	Registered nurses	General job satisfaction scale	0.89	Multivariate analysis; fixed effect analysis
				PES	0.75	
Spence Laschinger et al. (20)	Canada	Cross-sectional	Registered nurses	Job satisfaction scale	0.79	Multilevel structural, equation modeling techniques
				PES	0.83	
Ouyang et al. (21)	China	Cross-sectional	Registered nurses	Job satisfaction survey	0.91	Pearson correlations analysis; hierarchical multiple regression analysis
				PES	0.78	
Dahinten et al. (25)	Canada	Cross-sectional	Registered nurses	Revised MMSS 25-item	0.71–0.87	Pearson correlations analysis; hierarchical multiple regression analysis
				CWEQ-II	NA	
				PES	0.85–0.92	
Kretzschmer et al. (26)	USA	Cross-sectional	Registered nurses	CWEQ-II	0.70–0.95	Multiple regression analysis
				Job satisfaction survey	0.79–0.82	
Boamah et al. (27)	Canada	Mixed method	Registered nurses	Job satisfaction survey	0.92	Correlation analyses, multivariate regression analyses
				CWEQ-II	0.85	
Connally et al. (7)	Denmark	Mixed method	Registered nurses	PES	0.66	Correlation analyses, multivariate regression analyses
				CWEQ	0.40	
Lyden et al. (9)	USA	Cross-sectional	Registered nurses	Job satisfaction survey	0.79	Correlation analyses, multivariate regression analyses
				CWEQ-II	0.84	
García-Sierra et al. (8)	Spain	Cross-sectional	Registered nurses	CWEQ-II	0.91	Correlation analyses, multivariate regression analyses
de Almeida et al. (10)	Spain	Cross-sectional	Registered nurses	CWEQ-II	0.89	Correlation analyses, multivariate regression analyses
Choi et al. (28)	South Korea	Mixed method	Registered nurses	CWEQ-II	0.62–0.86	Correlation analyses, multivariate regression analyses

PES, psychological empowerment scale; CWEQ, conditions for work effectiveness questionnaire; WQI, work quality index; MMSS, Mueller/McCloskey nurse job satisfaction scale; NA, not available.

**Table 2:** Characteristics of the study population.

**Study included**	**Country**	**Sample size**	**Female, %**	**Age (years, mean ±SD)**	**Nursing experience (years, mean ±SD)**	**Response rate, %**
Morrison et al., (22)	USA	275	NA	NA	NA	64.00
Fuller et al. (23)	USA	230	NA	NA	NA	NA
Laschinger et al. (14)	Canada	404	NA	NA	NA	NA
Manojlovich et al. (15)	Canada	347	50.00	40.00 ± 8.07	16.00 ± 8.5.00	58.00
Larrabee et al. (2)	USA	90	93.30	34.60 ± 9.60	NA	NA
Laschinger et al. (3) ^a^	Canada	286	NA	NA	NA	NA
Laschinger et al. (3) ^b^	Canada	185	50	43.00 ^e^	19.00 ^e^	45.00
Laschinger et al. (12)	Canada	141	98.00	50.47 ± 7.56	29.22 ± 7.37	NA
Kostiwa et al. (24)	USA	56	89.00	42.32 ± 10.40	12.03 ± 7.77	63.00
Tourangeau et al. (31)	Canada	111	95.50	44.40 ± 11.60	7.80 ± 7.50	NA
Ahmad et al. (11) ^c^	Malaysia	388	99.00	32.74 ± 8.72	NA	NA
Ahmad et al. (11) ^d^	England	168	90.50	37.36 ± 10.32	NA	NA
Chang et al. (13)	China	330	100.00	NA	10.68 ± 8.51	66.00
Casey et al. (4)	Ireland	244	94.50	NA	NA	80.00
Engström et al. (5)	Sweden	46	100	49.20 ± 9.90	19.50 ± 8.90	NA
Sparks et al. (17)	USA	223	91.00	NA	NA	NA
Wagner et al. (18)	Canada	148	NA	NA	NA	31.00
Cramer et al. (30)	USA	84	NA	NA	NA	84.00
Spence Laschinger et al. (20)	Canada	545	95.80	42.00 ± 10.21	16.95 ± 10.86	40.00
Ouyang et al. (21)	China	726	94.21	NA	NA	85.40
Dahinten et al. (25)	Canada	1007	92.00	42.00 ± 11.00	>15.00	NA
Kretzschmer et al. (26)	USA	484	NA	NA	NA	63.70
Boamah et al. (27)	Canada	400	91.90	27.67 ± 6.88	1.17 ± 0.52	NA
Connolly et al. (7)	Denmark	112	NA	20.00–60.00 ^f^	NA	NA
Lyden et al. (9)	USA	142	83.00	NA	NA	50.00
Sierra et al. (8)	Spain	133	89.00	42.00 ± 10.00	17.00 ± 9.00	87.3
Almeida et al. (10)	Spain	151	87.40	44.04 ± 8.13	NA	58.75
Choi et al. (28)	South Korea	208	99.50	28.80 ± 5.40	7.30 ± 5.95	NA

SD, standard deviation; NA, not available.

a The study published by the Journal of Organizational Behavior.

b The study published by Nursing leadership.

c Study conducted in Malaysia.

d Study conducted in England.

e Mean.

f Range.

### Quality assessment

Supplementary Table 2 shows the results of the quality assessment. Seven studies were assessed as high quality (≥ 10 points), and 21 as medium (6–9 points) (Supplementary Table 2). All the included studies exhibited acceptable quality.

### Correlation between psychological empowerment and job satisfaction

**Figure 2:**
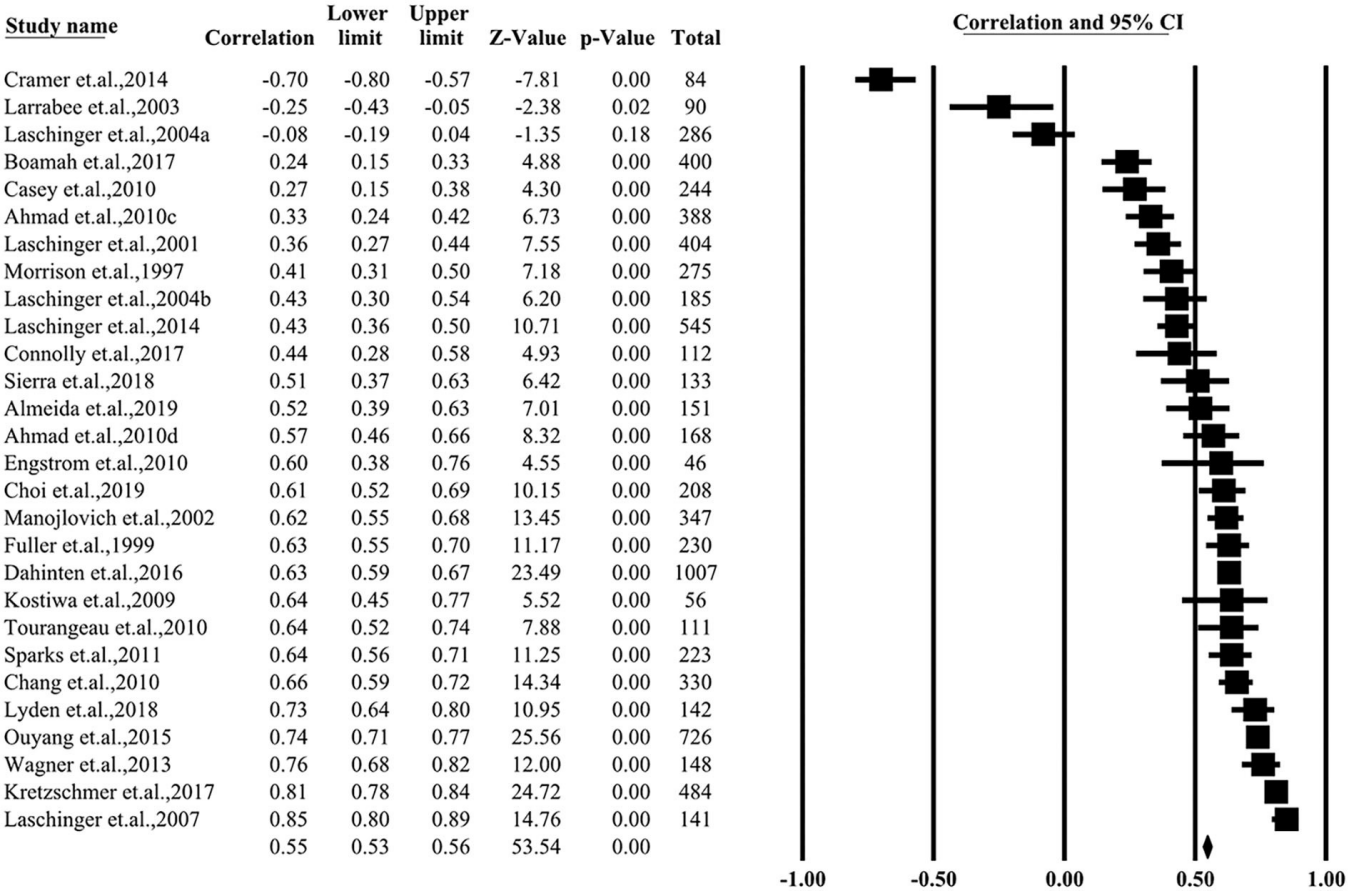
Summarized overall correlation.

The correlation between psychological empowerment and job satisfaction in the included studies varied largely. Two studies reported negative correlations (*r* < 0, *P* < 0.05) and four studies observed weaken correlations (0 < *r* < 0.4, *P* < 0.01). Five studies reported a strong correlation (*r* > 0.7, *P* < 0.01). The pooled *r* for all studies was 0.55 (95% CI = 0.53–0.56, *P* < 0.01) with a significant heterogeneity (*I*^2^ = 90%). The detailed data and funnel plot are depicted in Figure 2 and Supplementary Figure 1, respectively.

### Correlation between psychological empowerment and job satisfaction grouped by ethnicity

**Figure 3:**
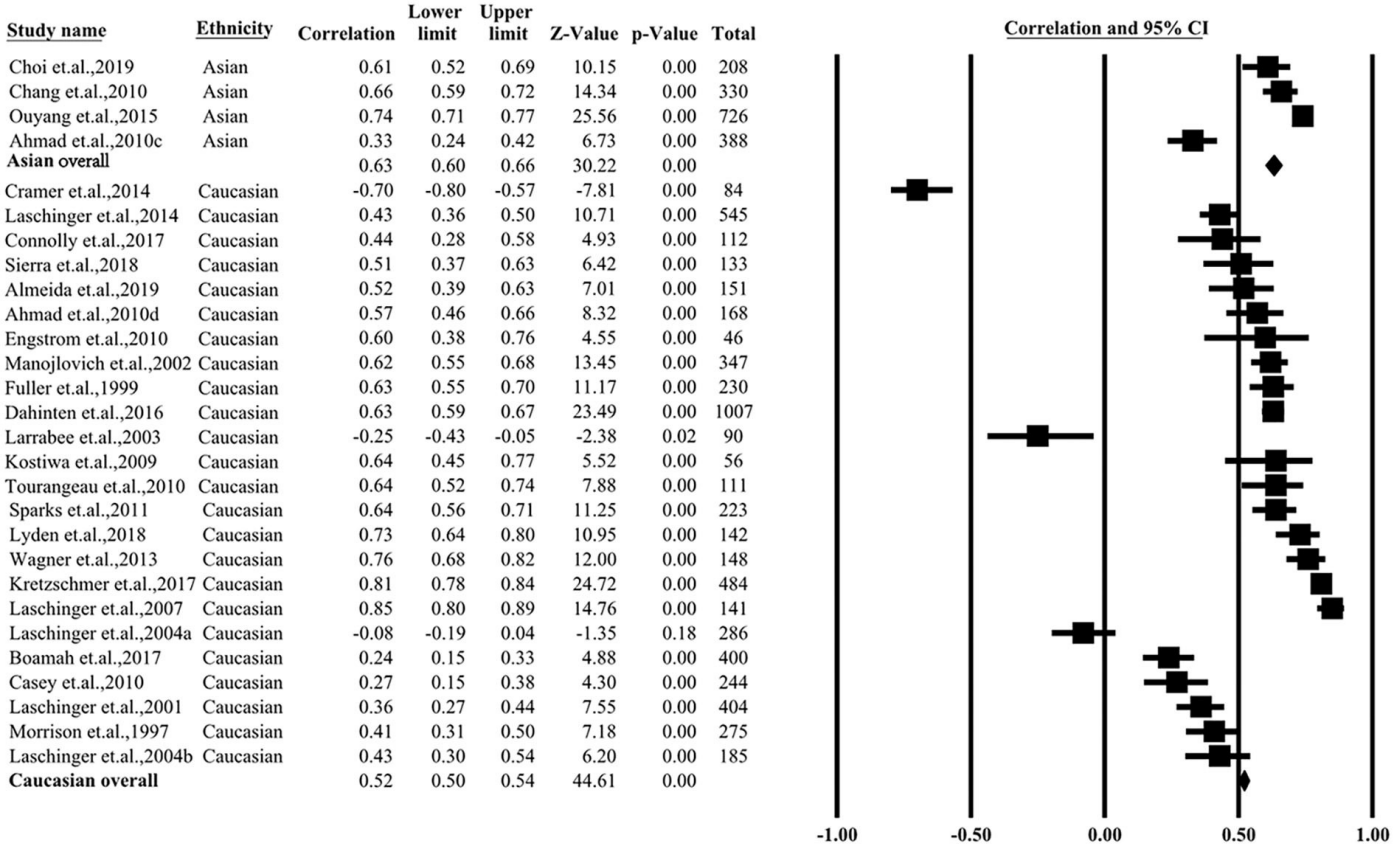
Summarized overall correlation categorized by ethnicity.

The studies from Canada (3, 12, 14–21), USA (2, 9, 19, 22–26), Spain (8, 10), England (11), Ireland (4), Denmark (7), and Sweden (5) were categorized into the Caucasian group. The studies conducted in South Korea (28), China (13, 27), and Malaysia (11) were categorized into the Asian group. When pooling the Caucasian group, the pooled *r* for all studies was 0.52 (95% CI = 0.50–0.54, *P* < 0.01, *I*^2^ = 89%). The pooled correlation among the Asian group presented a coefficient (*P* < 0.05) of 0.63 (95% CI = 0.60–0.66, *P* < 0.01, *I*^2^ = 88%). The data are graphically summarized in Figure 3.

### Exploration of heterogeneity

To explore the source of existing heterogeneity, we first pooled the studies by study design, cross-sectional method, and mixed method. The heterogeneities were found to be 90 and 89%, respectively. Then, the studies with high quality were pooled. As shown in Supplementary Figure 2, the pooled *r* for the studies with high-quality was 0.54 (95% CI = 0.41–0.64, *P* < 0.01). The heterogeneity (*I*^2^ = 61%) decreased to a moderate level. Additional analysis was carried out according to the sample size; however, high heterogeneity was observed.

### Publication bias

No potential publication bias was seen with *P*-values of Begg's rank correlation analysis and Egger's weighted regression analysis >0.05 (Supplementary Table 3).

## Discussion

### Main findings

In our meta-analysis, 28 studies with a total of 7,664 registered nurses were included for data analysis. All the included studies were of moderate or high quality. The correlation between psychological empowerment and job satisfaction was 0.55 (95% CI = 0.53–0.56, *P* < 0.01) irrespective of the source of the study population. Furthermore, the correlation among Asian group presented a slightly higher coefficient (*P* < 0.05) (*r* = 0.63) than that among the Caucasian group (*r* = 0.52).

### Comparison with previous studies

To date, one meta-analysis has investigated the association between psychological empowerment and job satisfaction in registered nurses (6). In this previous meta-analysis (6), the authors estimated the correlation between psychological empowerment and job satisfaction based on 11 eligible studies although a total of 20 studies were included finally. It showed that psychological empowerment and job satisfaction were significantly positively correlated (*p* < 0.001), but only moderately correlated (*r* = 0.353). Compared to the previous meta-analysis, the current meta-analysis revealed a strong correlation between psychological empowerment and job satisfaction (*r* = 0.55). Notably, the number of studies included in the current meta-analysis was 28, which was significantly more than that of the previous meta-analysis (*n* = 11). In addition, a significantly larger sample size of nurses was included (7,764 *vs*. 4,167). Undoubtedly, the current meta-analysis yielded more reliable and robust results than the previous meta-analysis. Moreover, the current meta-analysis also performed subgroup analyses to determine the correlation between psychological empowerment and job satisfaction from different population sources, providing more accurate results on this topic.

### Interpretation of findings

Psychological empowerment was deemed as a process that accompanied interaction between one's internal personal characteristics and the organization's environment. Specifically, psychological empowerment consists of four cognitive experiences (32): alignment between job requirements and beliefs, an individual's confidence in the ability to perform the activity skillfully, the sense of choice or control over one's work/autonomy and the commencement and maintenance of work activities in the workplace, and the sense of ability to influence important work outcomes. Previous studies concluded that the interaction between the external environment and inner individuality might be promoted by psychological empowerment (33, 34). Given the importance of psychological empowerment as an internal motivator, empowerment only has an impact when employees believe they are empowered (35). Studies have demonstrated that a high level of psychological empowerment is associated with lower stress, burnout and turnover intentions, higher organizational commitment, and job satisfaction (21, 36, 37). Furthermore, nurses who had structural empowerment were more likely to have positive beliefs about their capacity to contribute meaningfully to the workplace than nurses who did not have structural empowerment, and as a result, their job satisfaction increased (3, 16).

On the other hand, the current meta-analysis revealed that the correlation between psychological empowerment and job satisfaction in the Caucasian group was slightly lower than that in the Asia group. These inconsistent findings between the two groups might be explained according to the following reasons. First, nurses in various countries or regions have a diverse understanding of empowerment. In China, psychological empowerment is explained as a dynamic complementarity to prevent conflicts with authority figures, which might be influenced differently by Asian and Western cultures. Second, the working environments for nurses in Asian and Western countries varied markedly. Among the items assessing job satisfaction, supervision, nature of work, and communication were identified as crucial factors. The high workload and low compensation in Asian hospitals could be intervening obstacles in Asian studies, establishing a higher correlation between psychological empowerment and job satisfaction.

### Limitations

It is necessary to consider the limitations of the present study while interpreting the findings. First, most studies were cross-sectional, which might limit the ability to estimate causation and decrease the generalizability of the results. Second, a small number of studies were included, and a majority of them were conducted in Western countries and focused on the Caucasian population. The findings might be affected by environmental, medical level, and genetic factors, which can only partially annotate the associations, and the representativeness for the target population might be weakened. Moreover, the varied scales used in the included studies to examine both constructs of interest might also cause heterogeneity. Third, the measure of psychological empowerment and job satisfaction varied largely. A large proportion of the studies used widely applied methods. In addition, there are several definitions of psychological empowerment and job satisfaction. The definition of the targeted population is based on heterogeneity; also, it reduced the stability of the results. Fourth, the estimation of the reliability by Cronbach's coefficient varied according to the studies. Therefore, we could not correct attenuation, which might reflect the true effects. Finally, there were many intermediary factors affecting the relationship between psychological empowerment and job satisfaction; however, relevant data could be analyzed and summarized yet. Therefore, more studies are needed in the future to investigate the role of these intermediary factors.

## Conclusions

In this study, we summarized the correlations between psychological empowerment and job satisfaction and pooled results obtained from 28 studies. Next, we observed a significant correlation between psychological empowerment and job satisfaction. Moreover, the pooled correlation among the Asian group was slightly higher than that of the Caucasian group. Strategies to provide relief from psychological burdens would be beneficial to address mental exhaustion.

## Implications for practice and future studies

This meta-analysis will first assist administrators and hospitals in developing strategies for creating and maintaining an empowered workplace which will in turn increase job satisfaction among nurses and reduce turnover. Second, this study also suggests that additional research with larger sample sizes should be conducted in various counties to verify our findings. Finally, it is necessary to conduct more longitudinal, qualitative, and interventional studies to confirm the causal link between psychological empowerment and nurse job satisfaction.

## Data availability statement

The original contributions presented in the study are included in the article/Supplementary material, further inquiries can be directed to the corresponding author/s.

## Author contributions

Study design: LG. Data collection: LW and BP. Data analysis: LG, LW, and BP. Manuscript writing: LG and LW. All authors contributed to the article and approved the submitted version.

## Conflict of interest

The authors declare that the research was conducted in the absence of any commercial or financial relationships that could be construed as a potential conflict of interest.

## Publisher's note

All claims expressed in this article are solely those of the authors and do not necessarily represent those of their affiliated organizations, or those of the publisher, the editors and the reviewers. Any product that may be evaluated in this article, or claim that may be made by its manufacturer, is not guaranteed or endorsed by the publisher.
