# Supplementary material for: Psychological empowerment and job satisfaction in nurses: A systematic review and meta-analysis
Source: Front Public Health. 2022 Nov 11;10:1022823. doi: 10.3389/fpubh.2022.1022823 (PMC9692104; doi:10.3389/fpubh.2022.1022823)
Supplement: Supplementary file 1 [file Table_1.DOCX]

**Supplementary Materials**

**
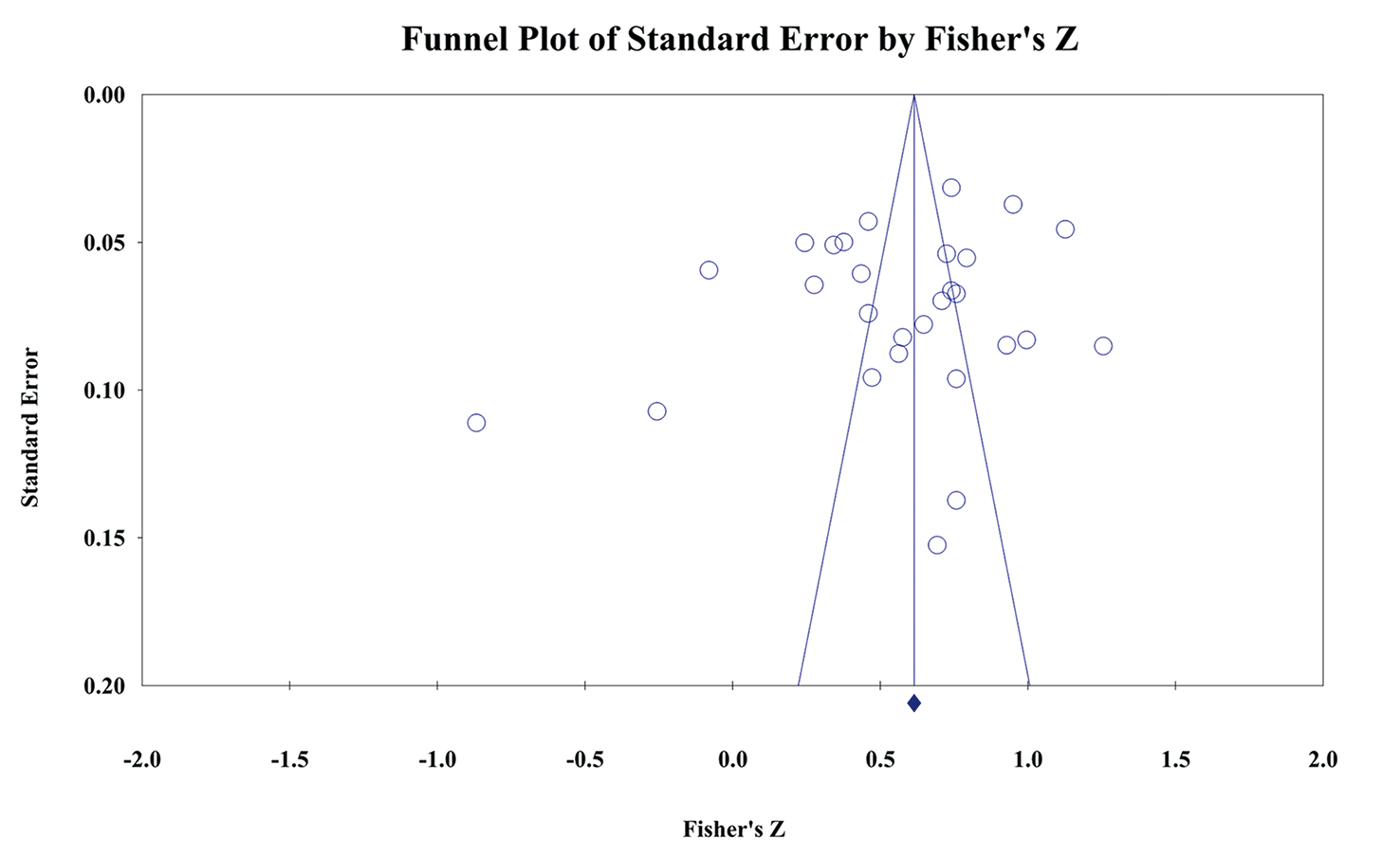
**

**Supplementary Figure 1. Funnel plot for overall correlation**

**
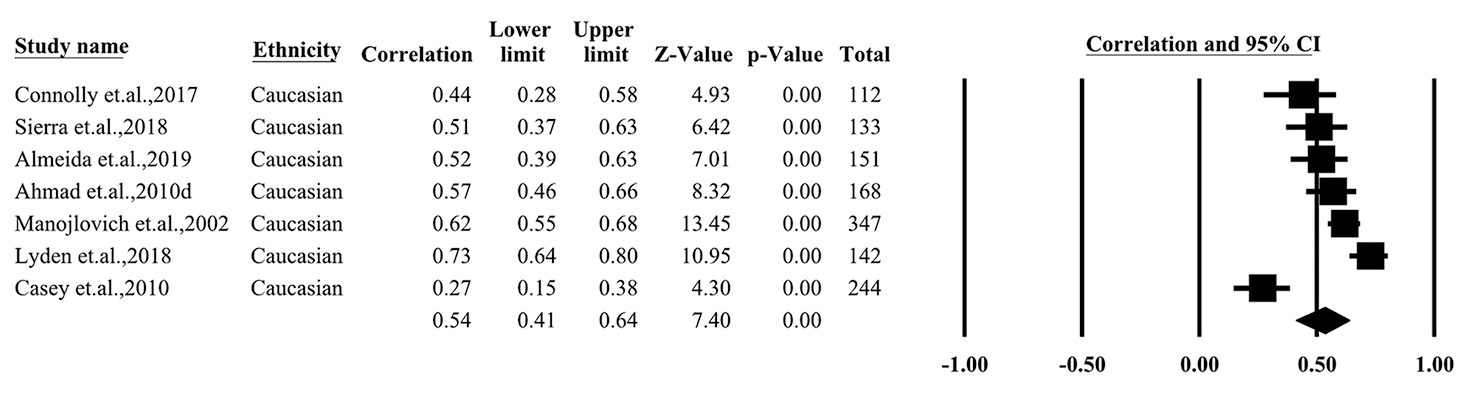
**

**Supplementary Figure 2. Summarized overall correlation for studies with high quality**

**Supplementary Table 1. Quality assessment and validity tool for correlational studies**

| **Study included** | **NO** | **Yes** |
| --- | --- | --- |
| 1. Was the study prospective? |  |  |
| 2. Was probability sampling used? |  |  |
| 3. Was sample size justified? |  |  |
| 4. Was sample drawn for more than one site? |  |  |
| 5. Was anonymity protected? |  |  |
| 6. Response rate was > 60%? |  |  |
| 7. Was the outcome measured reliably? |  |  |
| 8. Was the outcome measured using a valid instrument? |  |  |
| 9. Was the dependent variable measured using a valid instrument? |  |  |
| 10. If a scale was used for measuring the dependent variable, was the internal consistency ≥ 70? ^*^ |  |  |
| 11. Was a theoretical framework used for guidance? |  |  |
| 12. If multiple outcomes were studied, was the correlation analyzed? |  |  |
| 13. Were outliers managed? |  |  |

* ranges 0-2 points.

**Supplementary Table 2. Quality Assessment for each study**

| **Study included** | Prospective study | Probability sampling | Sample size justification | Multi-site sampling | Protection of anonymity | Response rate ≥ 60% | Reliable independent variable measurement | Valid instrument used for independent variable measurement | Valid instrument used for dependent variable measurement | Internal consistency ≥ 0.70 for dependent variable scale (if used) | Use of a theoretical framework | Analysis of correlations for multiple outcomes | Management of outliers | **Overall Score** |
| --- | --- | --- | --- | --- | --- | --- | --- | --- | --- | --- | --- | --- | --- | --- |
| Morrison et al., 1997 | 0 | 0 | 0 | 1 | 0 | 0 | 1 | 1 | 1 | 2 | 1 | 1 | 0 | 8 |
| Fuller et al., 1999 | 0 | 0 | 0 | 1 | 0 | 0 | 0 | 1 | 1 | 1 | 1 | 1 | 0 | 6 |
| Laschinger et al., 2001 | 0 | 0 | 0 | 1 | 0 | 0 | 1 | 1 | 1 | 2 | 1 | 1 | 0 | 8 |
| Manojlovich et al., 2002 | 0 | 0 | 0 | 1 | 1 | 1 | 1 | 1 | 1 | 2 | 1 | 1 | 0 | 10 |
| Larrabee et al., 2003 | 0 | 0 | 0 | 1 | 0 | 0 | 1 | 1 | 1 | 1 | 1 | 1 | 0 | 7 |
| Laschinger et al., 2004^a^ | 0 | 0 | 0 | 1 | 0 | 0 | 1 | 1 | 1 | 2 | 1 | 1 | 0 | 8 |
| Laschinger et al., 2004^b^ | 1 | 0 | 1 | 0 | 0 | 0 | 1 | 1 | 1 | 2 | 1 | 1 | 0 | 9 |
| Laschinger et al., 2007 | 0 | 0 | 0 | 1 | 0 | 0 | 1 | 1 | 1 | 1 | 1 | 1 | 0 | 7 |
| Kostiwa et al., 2009 | 0 | 0 | 0 | 1 | 0 | 0 | 1 | 1 | 1 | 2 | 1 | 1 | 0 | 8 |
| Tourangeau et al., 2010 | 0 | 0 | 0 | 1 | 0 | 0 | 1 | 1 | 1 | 2 | 1 | 1 | 0 | 8 |
| Ahmad et al., 2010 | 0 | 0 | 0 | 1 | 1 | 1 | 1 | 1 | 1 | 2 | 1 | 1 | 0 | 10 |
| Chang et al., 2010 | 0 | 0 | 0 | 1 | 0 | 0 | 1 | 1 | 1 | 2 | 1 | 1 | 0 | 8 |
| Casey et al., 2010 | 0 | 0 | 0 | 1 | 1 | 1 | 1 | 1 | 1 | 2 | 1 | 1 | 0 | 10 |
| Engstrom et al., 2010 | 0 | 0 | 1 | 1 | 0 | 0 | 1 | 1 | 0 | 1 | 1 | 0 | 0 | 6 |
| Sparks et al., 2011 | 0 | 0 | 0 | 1 | 1 | 0 | 1 | 1 | 1 | 2 | 1 | 1 | 0 | 9 |
| Wagner et al., 2013 | 0 | 0 | 0 | 1 | 0 | 0 | 1 | 1 | 1 | 2 | 1 | 1 | 0 | 8 |
| Cramer et al., 2014 | 0 | 0 | 1 | 1 | 0 | 0 | 1 | 1 | 0 | 1 | 0 | 1 | 0 | 6 |
| Laschinger et al., 2014 | 0 | 0 | 0 | 1 | 1 | 0 | 1 | 1 | 1 | 2 | 1 | 1 | 0 | 9 |
| Ouyang et al., 2015 | 0 | 0 | 0 | 1 | 0 | 1 | 1 | 1 | 1 | 2 | 1 | 1 | 0 | 9 |
| Dahinten et al., 2016 | 0 | 0 | 0 | 1 | 0 | 0 | 1 | 1 | 1 | 2 | 1 | 1 | 0 | 8 |
| Kretzschmer et al., 2017 | 1 | 0 | 1 | 0 | 0 | 0 | 1 | 1 | 1 | 2 | 1 | 1 | 0 | 9 |
| Boamah et al., 2017 | 0 | 0 | 0 | 0 | 1 | 0 | 1 | 1 | 1 | 2 | 1 | 1 | 0 | 8 |
| Connolly et al., 2017 | 0 | 0 | 1 | 1 | 0 | 1 | 1 | 1 | 1 | 2 | 1 | 1 | 0 | 10 |
| Lyden et al., 2018 | 0 | 0 | 0 | 1 | 1 | 1 | 1 | 1 | 1 | 2 | 1 | 1 | 0 | 10 |
| Sierra et al., 2018 | 0 | 0 | 1 | 1 | 0 | 1 | 1 | 1 | 1 | 2 | 1 | 1 | 0 | 10 |
| Almeida et al., 2019 | 0 | 0 | 1 | 1 | 0 | 1 | 1 | 1 | 1 | 2 | 1 | 1 | 0 | 10 |
| Choi et al., 2019 | 0 | 0 | 0 | 1 | 0 | 0 | 1 | 1 | 1 | 2 | 1 | 1 | 0 | 8 |

^a^, the study published by Journal of Organizational Behavior.

^b^, the study published by Nursing leadership.

**Supplementary Table 3. Publication bias of summarized outcomes**

| **Outcomes** | **Begg (*P*-value)** | **Egger (*P*-value)** |
| --- | --- | --- |
| Summarized overall correlation | 0.59 | 0.23 |
| Summarized overall correlation for Asian | 0.73 | 0.52 |
| Summarized overall correlation for Caucasian | 0.86 | 0.41 |
| Summarized overall correlation for high-quality studies | 0.70 | 0.44 |
